# Proteomics in Biomarker Discovery for Tuberculosis: Current Status and Future Perspectives

**DOI:** 10.3389/fmicb.2022.845229

**Published:** 2022-04-26

**Authors:** Jiubiao Guo, Ximeng Zhang, Xinchun Chen, Yi Cai

**Affiliations:** ^1^College of Pharmacy, Shenzhen Technology University, Shenzhen, China; ^2^Guangdong Provincial Key Laboratory of Regional Immunity and Diseases, Department of Pathogen Biology, School of Medicine, Shenzhen University, Shenzhen, China

**Keywords:** tuberculosis, proteomics, biomarker, diagnosis, mass spectrometry

## Abstract

Tuberculosis (TB) continues to threaten many peoples’ health worldwide, regardless of their country of residence or age. The current diagnosis of TB still uses mainly traditional, time-consuming, and/or culture-based techniques. Efforts have focused on discovering new biomarkers with higher efficiency and accuracy for TB diagnosis. Proteomics—the systematic study of protein diversity—is being applied to the discovery of novel protein biomarkers for different types of diseases. Mass spectrometry (MS) technology plays a revolutionary role in proteomics, and its applicability benefits from the development of other technologies, such as matrix-based and immune-based methods. MS and derivative strategies continuously contribute to disease-related discoveries, and some promising proteomic biomarkers for efficient TB diagnosis have been identified, but challenges still exist. For example, there are discrepancies in the biomarkers identified among different reports and the diagnostic accuracy of clinically applied proteomic biomarkers. The present review summarizes the current status and future perspectives of proteomics in the field of TB biomarker discovery and aims to elicit more promising findings for rapid and accurate TB diagnosis.

## Introduction

Tuberculosis (TB), caused by the pathogenic bacteria *Mycobacterium tuberculosis* (*Mtb*), continues to be a leading public health threat that affects all countries and age groups (Zumla et al., 2013). In 2020, approximately 10 million people developed TB, and 1.3 million people died from the infection worldwide. Of increasing concern, a total of 157,903 drug-resistant cases were reported, with 132,222 cases of multidrug- or rifampicin-resistant TB and 25,681 cases of pre-extensively drug-resistant TB or extensively drug-resistant TB detected, although this was a large fall (of 22%) from the total of 201,997 people detected with drug-resistant TB in 2019 (WHO, 2021). Tuberculosis control strategies aim to reduce the spread of the infection and cure infectious TB patients, who need rapid and accurate diagnosis to facilitate the administration of prompt anti-TB treatment, thereby helping to reduce the transmission of TB and development of drug resistance.

*Mtb* is an intracellular pathogen that preferentially infects host macrophages and primarily resides within lung granulomas (Brites and Gagneux, 2015; Elkington et al., 2021), making directly detecting *Mtb* in human body fluids, such as blood, saliva, or urine, impossible. A current challenge in TB diagnosis is the development of rapid point-of-care tests. Sputum smear microscopy is the most common way of diagnosing TB worldwide (Steingart et al., 2006; Molicotti et al., 2014); however, the sensitivity of sputum smear microscopy ranges from 30 to 60% and is largely dependent on the operator and the abundance of *Mtb* in the samples (Molicotti et al., 2014). Microbiological culture is considered a diagnostic gold standard, but it requires several weeks and laboratory containment facilities to culture and identify *Mtb* in samples due to the slow growth rate and biohazard level of the microbe (Lagier et al., 2015). The rapid molecular-based diagnostic test Xpert MTB/RIF has high sensitivity and specificity for the diagnosis of smear-positive sputum TB patients (Steingart et al., 2013); however, Xpert MTB/RIF has a lower sensitivity for smear-negative sputum samples, leaving the diagnosis of a significant proportion of TB-infected cases reliant on diagnostic tests with sub-optimal accuracy.

Recently, many researchers have focused on the discovery of host biomarkers for TB diagnostics. Host immune responses to *Mtb* infection (Dey and Bishai, 2014) leave traceable signals within the host that may prove valuable for the accurate diagnosis and/or prognosis of TB (Parida and Kaufmann, 2010). Increasingly, investigators are validating the feasibility and accuracy of using proteomic signatures for TB diagnosis and prognosis (De Groote et al., 2017b; Penn-Nicholson et al., 2019). For example, our team previously screened for and validated key proteomic TB biomarkers using an antibody-based array for whole-blood samples that were stimulated with pooled *Mtb* peptides (ESAT-6 and CFP-10 derived peptides) (Chen et al., 2009) or mitogen, and we successfully identified an eight-protein bio-signature of I-TAC, I-309, MIG, granulysin, FAP, MEP1B, furin, and LYVE-1. The combination of the eight biomarkers allowed us to distinguish TB from healthy control individuals in a test cohort with a specificity and sensitivity of 83% (95% CI, 71–91%) and 76% (95% CI, 56–90%), respectively (Yang et al., 2020). In this review, we summarize the proteomics research approaches followed over the past several years, focusing on the progress of TB biomarker discovery via proteomics, with the hope of eliciting more promising biomarker discoveries for rapid and accurate TB diagnosis.

## Proteomics Research Approaches

Proteomics is the systematic study of the proteome with the aim of uncovering the nature of sophisticated protein-interaction networks with respect to protein expression, structure, function, and control of biological processes within an organism (Patterson and Aebersold, 2003). By comparing different patterns within the proteome, proteomics has continued to provide a powerful method for studying the changes in protein diversity that accompany health and disease processes, making the clinical diagnosis, prognosis, and even treatment of different diseases possible (Kavallaris and Marshall, 2005).

**FIGURE 1:**
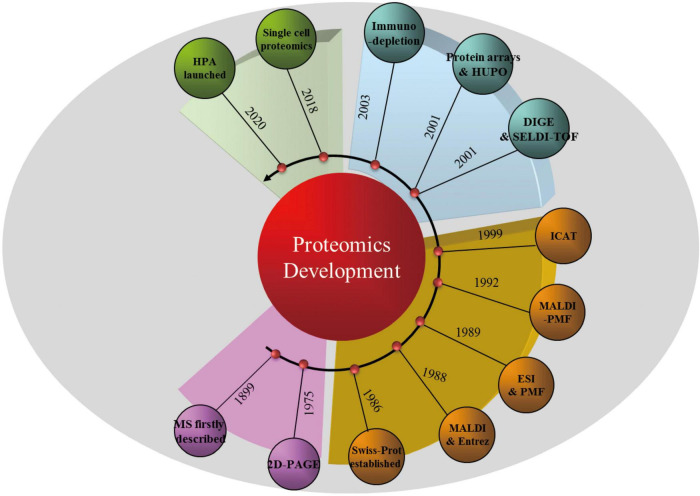
Timeline of proteomic technologies and protein database development. MS, mass spectrometry; 2D-PAGE, two-dimensional polyacrylamide gel electrophoresis; MALDI, matrix-assisted laser desorption/ionization; ESI, electrospray ionization; PMF, peptide mass fingerprinting; ICAT, isotope-coded affinity tag; DIGE, difference-gel 2D-electrophoresis; SELDI-TOF, surface-enhanced laser desorption/ionization time of flight; HUPO, Human Proteome Organization; HPA, Human Protein Atlas.

It is the development of technology and informatics that make a new concept achievable, as is the case for proteomics. Figure 1 illustrates the timeline of proteomic technology and protein database development that has promoted proteomics research. However, the proteome of a particular cell type or tissue is a mixture of all the proteins expressed (Wilkins, 2009); thus, protein separation is a prerequisite for single-protein analysis. As early as 1975, two-dimensional polyacrylamide gel electrophoresis (2D-PAGE), which separates proteins based on their size and surface charge, was first employed to separate ribosomal proteins of *Escherichia coli* (Knopf et al., 1975). Mass spectrometry (MS), which is usually applied to the identification of individual protein spots separated by 2D-PAGE, was first described in 1899. It was not, however, until the development of non-destructive large-biomolecule ionization methods, including matrix-assisted laser desorption/ionization (MALDI) (Karas and Hillenkamp, 1988) and electrospray ionization (Fenn et al., 1989), that the widespread application of MS became feasible. Peptide mass fingerprinting (Henzel et al., 2003) and isotope-coded affinity tag (Gygi et al., 1999) approaches were then combined with different MS strategies for the accurate quantification of concurrent peptides from proteins that are found abundantly or even in trace amounts in complex mixtures.

Benefiting from the rapid development of technology during the 21st century, some classical technologies have helped to further revolutionize proteomics. For example, 2D-PAGE and MS have been developed into techniques such as difference-gel 2D-electrophoresis (Larbi and Jefferies, 2009), surface-enhanced laser desorption/ionization time of flight (SELDI-TOF) MS (Marcos et al., 2013), quantitative chemical cross-linking with MS (Wippel et al., 2021), and immunodepletion techniques for “low-abundance” protein determination (Liu et al., 2021b). Moreover, “protein chips” have been designed and combined with MS approaches for the specific analysis of selected proteins (Zhou et al., 2001). It is important to consider that, coupled with proteomic technologies, proteomics databases, including Swiss-Prot, Entrez, and the Human Proteome Organization, also continue to contribute to the development of proteomics (Kavallaris and Marshall, 2005). Moreover, in addition to other single-cell approaches (Mauger et al., 2021), single-cell proteomics is emerging as a way to identify and quantify the proteins in individual cells (Li et al., 2021). Although there are no high-throughput proteomic “sequencers” available as yet, it is believed that this technology will contribute to the discovery of new aspects of protein and cell biology in the future.

## Diagnostic Application of Proteomic Biomarkers for Tuberculosis

Upon *Mtb* infection, the host cells’ response is to produce and secrete certain effectors to deal with the invading bacteria (Dey and Bishai, 2014; Brites and Gagneux, 2015; Llibre et al., 2021). Blood is the primary vehicle for the transport of either host effectors or *Mtb* factors and is usually routinely sampled for clinical testing, making blood samples readily accessible for research purposes and diagnostic tests. The recent advances in proteomics make the simultaneous detection of thousands of proteins possible, facilitating the capture of effectors and thus paving the way for alternative and efficient TB biomarker discovery. In this section, we summarize the recent developments in the identification of proteomic biomarkers that can be used for differentiating TB from healthy or other disease status individuals. This has been achieved by categorizing the origin of the biomarkers from either blood samples, urine samples, or other types of human body fluids.

### Identification of Tuberculosis Biomarkers in Human Blood Samples

Human blood and derivative samples are ideal for TB diagnosis with respect to convenience, feasibility, and the amount of sample that can be collected. In addition, upon *Mtb* infection, molecules secreted during host immune responses, such as cytokines, are mainly delivered via the blood. The advantages of blood samples make them a priority choice, and most studies of proteomic biomarkers for TB diagnosis are based on blood samples. Some biomarkers, for example, ESAT6 (Chiappini et al., 2012; Liu et al., 2017), IP-10 (Estevez et al., 2020), and CD161 (Yang et al., 2015), are secreted by either *Mtb* or the host and have been verified and validated for the detection of *Mtb* and TB diagnosis. But due to the low chance of detecting *Mtb* traces in blood samples and the reported inconsistencies among different research findings, more intensive investigations are necessary in the search for further potential biomarkers for TB diagnosis.

Agranoff et al. (2006) undertook research to discriminate between TB-infected and control individuals by utilizing SELDI-TOF MS (von Eggeling et al., 2001; Issaq et al., 2002) to search for proteomic biomarkers in serum samples. In the first phase of the study, they recruited 349 individuals and studied 179 confirmed culture-positive TB samples and 170 controls collected at St George’s Hospital, United Kingdom; Angola; the Gambia; and Uganda. After profiling all the serum samples with weak cation exchange (CM10) protein chip arrays and supervised machine learning classification methods, the authors chose a Gaussian kernel support vector machine classifier to discriminate the proteomic profile of patients with active TB from that of controls, and the diagnosis sensitivity was 93.5%, specificity was 94.9%, and overall diagnostic accuracy of 94% as the best discriminator for the TB and control groups. A second independent and prospectively collected testing set that included 41 validation samples (18 TB and 23 controls) achieved a sensitivity of 88.9% and specificity of 77.2%. This study was robust but did not clarify the identity of the potential protein biomarkers, which hampered their subsequent application in other sets of samples. In addition, the size of the second testing set was limited, and a larger set of testing samples would have provided a more accurate validation.

Another study (De Groote et al., 2017b) reported the execution of a large multi-center study designed to search for a diagnostic serum protein signature for pulmonary TB. Using the 4,000-plex SOMAscan assay (De Groote et al., 2017a), they performed in-depth proteomic analysis of 1,470 serum samples from seven TB-endemic countries: South Africa, Peru, Zimbabwe, Uganda, Vietnam, Colombia, and Bangladesh. A total of 504 samples (252 non-TB and 252 TB) were tested on SOMAscan for biomarker discovery. The identified HR6 model of host response markers, which included SYWC, kallistatin, complement C9, gelsolin, testican-2, and aldolase C, was subjected to testing with a blinded verification set of 204 samples and reached an area under the curve (AUC) of 0.87 (95% CI, 0.81–0.91). Besides the identified HR6 protein signature, several previously described TB markers, such as IP-10, LBP, FCG3B, and TSP4, were also detected in this study. The reason that IP-10 and other known markers were not included in the HR6 signature may be the statistical methods applied or other reasons. Although there were claims that the 4,000-plex SOMAscan assay is able to simultaneously measure > 4,000 proteins in serum samples, many other proteins, and thus other more promising biomarkers, could have been missed.

Tuberculosis (TB) co-infection is a leading cause of death among people living with HIV, and diagnostic biomarker discovery among this group of people is necessary to reduce mortality (Corbett et al., 2003). Singer et al. (2021) analyzed and compared plasma host proteins from subjects from South Africa (*n* = 30, representing a region of high TB incidence) and the United States (*n* = 24, representing a region of low TB incidence), and CD14, A2GL, NID1, SCTM1, and A1AG1 were identified as overlapping between both cohorts. The authors further assessed the diagnostic performance of these host proteins using cross-validation and found that panels of 5–12 proteins had excellent accuracy 0–6 (AUC 0.93) at 6–12 months (AUC 0.86) prior to TB diagnosis for the South African cohort and good accuracy 0–6 (AUC 0.74) at 6–12 months (AUC 0.76) prior to TB diagnosis for the United States cohort. In addition, Shen et al. (2020) analyzed 200 HIV-positive plasma samples using data-independent acquisition MS-based proteomics, and they reported that, in combination, the proteins markers AMACR, LDHB, and RAP1B may serve as TB markers for HIV-infected patients (Shen et al., 2020). Overall, the TB diagnosis of patients co-infected with HIV is difficult due to interference from the HIV infection. Intensive studies of large cohorts with diverse genetic backgrounds are needed to achieve accurate and consistent data.

Recently, an increasing number of investigations have reported new proteomic biomarkers for TB diagnosis using either blood, urine, or other body fluid samples (Table 1). It should be noted that discrepancies exist among the different investigation outcomes, even among similar studies (Table 1). In addition, most of the proteomic biomarkers identified in various body fluid samples originated from preliminary investigations, and there is still a long journey ahead before these potential biomarkers can be applied in clinical diagnosis. Moreover, due to the intracellular survival and other adaptation features of the bacterium (Brites and Gagneux, 2015), few studies have discovered mycobacterium-derived proteomic biomarkers that have the potential to be used in the diagnosis of active TB (Hendrickson et al., 2000; Chen et al., 2018). Some additional identified blood-based proteomic biomarkers are summarized in Table 1.

**TABLE 1:** Proteomic biomarker identification for TB diagnosis.

Sample type/size	Proteomic biomarker	Sensitivity and specificity	Technology employed	References
Total 196 urine samples	Glutathione peroxidase 3, neurotrimin, poliovirus receptor, signaling lymphocytic activation molecule family 1, and hemicentin-2	82.7% sensitivity and 92.3% specificity	LC–MS/MS	Liu et al. (2021a)
Total 342 plasma samples	CFHR5, LRG1, CRP, LBP, and SAA1	AUC of 0.93 (95% CI: 0.89–1.00, p ≤ 0.001) or 0.81 (95% CI: 0.68–0.94, p = 0.001)	High-resolution MS	Garay-Baquero et al. (2020)
Total 120 serum samples	sCD14, PGLYRP2, and FGA	AUC of 0.934, sensitivity of 81.2%, and specificity of 90%	MS strategy	Chen et al. (2020)
Total 6,363 plasma samples	Complement factor 9, IGFBP-2,CD79A, MXRA-7, NrCAM, CK-MB, and C1qTNF3/CTNFF3	AUC of 0.66 (0.56–0.75) or 0.65 (0.55–0.75)	Multiplexed proteomic assay (SOMAscan)	Penn-Nicholson et al. (2019)
1,470 serum samples	SYWC, kallistatin, complement C9, gelsolin, testican-2, and aldolase C	AUC of 0.95 or 0.92 in a blinded verification set	4,000-plex SOMAscan assay	De Groote et al. (2017b)
172 serum and plasma proteins	CLEC3B, ECM1, IGFALS, IGFBP3, SELL, and VWF	AUC > 0.85	MRM-MS assay	Bark et al. (2017)
Total 63 urine samples	IGKC, RBP4, PTGDS, AMBP, ORM1, IGCL2, and SECTM1	Not available	LC–MS/MS	Young et al. (2014)
Total 132 serum samples	Apolipoprotein CII (APOCII), CD5 antigen-like (CD5L), and retinol-binding protein 4 (RBP4)	93.42% sensitivity and 92.86% specificity	iTRAQ-coupled 2D LC–MS/MS technique	Xu et al. (2014)
Total 103 sputum samples	UqhC	Not available	2D-PAGE and MALDI-TOF/TOF MS	Fu et al. (2012)
Two isolated *Mtb* strains	Rv0443, Rv0379, and Rv0147	Not available	2D-PAGE and MS	Hadizadeh Tasbiti et al. (2021)
Total 285 urine samples	miR-625-3p, mannose-binding lectin 2, and inter-α-trypsin inhibitor H4	85.87% sensitivity and 87.50% specificity	2D-PAGE and MALDI-TOF/TOF MS	Wang et al. (2018)
Total 104 saliva samples	Salivary CRP, ferritin, serum amyloid P, MCP-1, alpha-2-macroglobulin, fibrinogen, and tissue plasminogen activator	78.1% sensitivity and 83.3% specificity	Luminex multiplex immunoassay	Jacobs et al. (2016)
Total 141 serum samples	2,024, 8,007, and 8,598 Da identified by biomarker patterns software	Blind test data indicated sensitivity of 80.0% and specificity of 84.2%	SELDI-TOF MS and protein-chip	Liu et al. (2011)
Total 264 serum samples	Three protein peaks at m/z 5,643, 4,486, and 4,360 Da	96.9% sensitivity, 97.8% specificity, and up to 97.3% accuracy	SELDI-TOF MS and protein-chip	Zhang et al. (2012)
Total 630 stimulated blood samples	I-TAC, I-309, MIG, granulysin, FAP, MEP1B, furin, and LYVE-1	For prediction cohort, specificity of 84% (95% CI 74–92%) and sensitivity of 75% (95% CI 57–89%)	Protein arrays	Yang et al. (2020)
Total 160 serum samples	S100A9, SOD3, and MMP9	Sensitivity of 92.5% and specificity of 95% for discriminating between TB and HC	iTRAQ-coupled 2D LC–MS/MS	Xu et al. (2015)
Total 391 serum samples	2,554.6, 4,824.4, 5,325.7, and 8,606.8 Da	Sensitivity of 83.3% and specificity of 84.2%	SELDI-TOF MS	Liu et al. (2013)
Total 390 serum samples	Serum amyloid A and transthyretin	Sensitivity of 88.9% and specificity of 77.2% in test cohort	SELDI-TOF MS and protein chip arrays	Agranoff et al. (2006)

*MRM-MS, mass spectrometry coupled with multiple-reaction monitoring; MS, mass spectrometry; LC–MS/MS, liquid chromatography coupled with tandem mass spectrometry; AUC, area under the curve; iTRAQ, isobaric tags for relative and absolute quantification; SELDI-TOF, surface-enhanced laser desorption/ionization time of flight; 2D-PAGE, two-dimensional polyacrylamide gel electrophoresis; MALDI-TOF/TOF MS, matrix-assisted laser desorption/ionization-time of flight tandem mass spectrometry; TB, tuberculosis; HC, healthy controls.*

### Identification of Tuberculosis Biomarkers in Human Urine Samples

Besides blood samples, urine samples are another common choice for identifying proteomic biomarkers for human TB diagnosis, and some relevant investigations have been reported. In 2021, Liu et al. (2021a) analyzed and compared the urinary proteomic profiles of TB patients and healthy controls. They first screened for potential biomarkers using the liquid chromatography coupled with tandem mass spectrometry (LC–MS/MS) technique on 20 TB and 20 healthy control samples, then further validated the identified proteomic biomarkers in another 52 TB, 52 latent tuberculosis infection (LTBI), and 52 healthy control samples. Based on the data, they concluded that a combination of glutathione peroxidase 3 (P22352), neurotrimin (Q9P121), poliovirus receptor (P15151), signaling lymphocytic activation molecule family 1 (Q13291), and hemicentin-2 (Q8NDA2) could potentially be applied to the diagnosis of TB, with an 82.7% sensitivity for TB diagnosis and a 92.3% specificity for the diagnosis of TB in the LTBI category. By comparing urine samples from 21 active TB, 24 LTBI, and 18 healthy controls via LC–MS/MS, Young et al. identified IGKC, RBP4, PTGDS, AMBP, ORM1, IGCL2, and SECTM1 as potential protein biomarkers for distinguishing TB from LTBI or healthy control samples. However, they did not validate the group of biomarkers in a second cohort and did not clarify the diagnostic sensitivity or specificity (Young et al., 2014). In addition, a unique 21-mer *Mtb* peptide sequence (VVLGLTVPGGVELLPGVALPR) was identified (Pollock et al., 2018) from urine samples of Zimbabwean patients that showed 95% sequence homology with *Mtb* oxidoreductase (MRGA423_21210) from the clinical isolate MTB423 (identified in Kerala, India), but the relevance of this occasionally identified *Mtb*-originating protein biomarker needs to be verified in further investigations.

### Identification of Tuberculosis Biomarkers in Other Human Body Fluids

Saliva and sputum, which contain thousands of proteins, mRNA, and bacterial species, have been used widely for biomarker studies and as samples for disease diagnosis and assessment (Ruhl, 2012; Carpenter, 2013; Kaczor-Urbanowicz et al., 2017; Sun et al., 2017). Collecting a saliva/sputum sample is easy, non-invasive, and more acceptable for repeat testing. Several biomarkers for the diagnosis of TB have been identified in saliva by proteomics approaches. Recently, P01011, Q8NCW5, P28072, A0A2Q2TTZ9, and Q99574 were identified using a QExactive Orbitrap mass spectrometer, with which the combined five-protein bio-signature was shown, after leave-one-out cross validation, to yield an AUC of 1.00 (95% CI, 1.00–1.00), sensitivity of 100% (95% CI, 76.2–100%), and specificity of 90.9% (95% CI, 58.7–99.8%) in TB diagnosis (Mutavhatsindi et al., 2021). Using MS, a signature comprising β-integrin, vitamin D-binding protein, uteroglobin, profilin, and cathelicidin antimicrobial peptide in saliva was confirmed to differentiate active TB patients from non-TB patients with an AUC of 0.75 (Bishwal et al., 2019). Mariam and colleagues investigated the sputum proteome of patients with active and latent TB infections as well as community controls using an ultrafast sample-preparation approach (HaileMariam et al., 2018). A 49-protein signature was used to successfully distinguish TB from control subjects; however, this panel of proteins was unable to differentiate LTBI from healthy subjects. In another study, salivary proteomic analysis demonstrated that TB patients exhibit a specific accumulation of proteins related to complement activation, inflammation, and the modulation of the immune response and a decrease in proteins related to glucose and lipid metabolism. A group of proteins, including haptoglobin, alpha-1-acid glycoprotein 1 and 2, immunoglobulin gamma 4 chain, fibrinogens, dermcidin, protein disulfide isomerase, triosephosphate isomerase, and ras GTPase-activating-like protein, are other potential biomarkers for the diagnosis of TB (Mateos et al., 2019).

## Challenges and Future Perspectives

Intensive investigations into *Mtb–*host interactions have broadened and deepened our knowledge on the strategies applied by *Mtb* to infect hosts and the host responses upon infection. The development of technologies, especially MS, has facilitated proteomic biomarker identification with high efficiency, but there are still some technical challenges slowing down the application of proteomics to the diagnosis of active TB. Sampling complexities (e.g., the timing of sample collection, small-molecule interference, and the coexistence of TB with other types of disease), differences in protein abundances, the presence of isoforms, and post-translational modifications are barriers preventing the identification of accurate and universal biomarkers with current technologies and strategies. More powerful techniques with improved sensitivity will be required, especially for low-abundance proteins and for differentiating protein isoforms and modifications. In addition, there is a risk of diagnostic bias when using a single protein biomarker, and a group of biomarkers within a panel could be detected simultaneously using current technologies, but discrepancies among different reports are increasing, providing confusing or even misleading evidence for the optimum TB diagnostic markers. One reason for the unsatisfactory reproducibility of the discovered biomarkers is that the studied cohorts have differed with regard to their genomic backgrounds, immune responses, or other factors. Large cohorts of samples from various populations that include a diversity of TB patient statuses may be needed to overcome this problem. Moreover, a combination of different “omics” techniques may be useful for improving TB diagnostic accuracy and consistency, and single-cell proteomics holds particular promise as a method that will advance active TB diagnosis in the future (Petelski et al., 2021).

## Author Contributions

JG and YC contributed to the conceptualization and design, reviewed the literature, prepared the figures and tables, and wrote the original draft of the manuscript. XZ contributed to literature review and data preparation. XC and YC supervised the data and wrote, reviewed, and edited the manuscript. JG, XC, and YC contributed to the project administration, funding acquisition, and resources. All authors contributed to the article and approved the submitted version.

## Conflict of Interest

The authors declare that the research was conducted in the absence of any commercial or financial relationships that could be construed as a potential conflict of interest.

## Publisher’s Note

All claims expressed in this article are solely those of the authors and do not necessarily represent those of their affiliated organizations, or those of the publisher, the editors and the reviewers. Any product that may be evaluated in this article, or claim that may be made by its manufacturer, is not guaranteed or endorsed by the publisher.
